# Genetical genomics of *Populus* leaf shape variation

**DOI:** 10.1186/s12870-015-0557-7

**Published:** 2015-06-30

**Authors:** Derek R. Drost, Swati Puranik, Evandro Novaes, Carolina R.D.B. Novaes, Christopher Dervinis, Oliver Gailing, Matias Kirst

**Affiliations:** School of Forest Resources and Conservation, University of Florida, P.O. Box 110410, Gainesville, FL 32611 USA; Plant Molecular and Cellular Biology Graduate Program, University of Florida, P.O. Box 110690, Gainesville, FL 32611 USA; School of Forest Resourse and Environmental Sciences, Michigan Technological University, Houghton, MI 49931 USA; University of Florida Genetics Institute, University of Florida, P.O. Box 103610, Gainesville, FL 32611 USA; Seminis, Inc., 37437 State Highway 16, Woodland, CA 95695 USA; Escola de Agronomia, Universidade Federal de Goiás, Rodovia Goiânia/Nova Veneza, Km0 - Caixa Postal 131, Goiânia, GO 74690-900 Brazil

**Keywords:** Leaf morphology, QTL analysis, Expression QTL, Genomics, *Populus trichocarpa*, ADP-ribosylation factor

## Abstract

**Background:**

Leaf morphology varies extensively among plant species and is under strong genetic control. Mutagenic screens in model systems have identified genes and established molecular mechanisms regulating leaf initiation, development, and shape. However, it is not known whether this diversity across plant species is related to naturally occurring variation at these genes. Quantitative trait locus (QTL) analysis has revealed a polygenic control for leaf shape variation in different species suggesting that loci discovered by mutagenesis may only explain part of the naturally occurring variation in leaf shape. Here we undertook a genetical genomics study in a poplar intersectional pseudo-backcross pedigree to identify genetic factors controlling leaf shape. The approach combined QTL discovery in a genetic linkage map anchored to the *Populus trichocarpa* reference genome sequence and transcriptome analysis.

**Results:**

A major QTL for leaf lamina width and length:width ratio was identified in multiple experiments that confirmed its stability. A transcriptome analysis of expanding leaf tissue contrasted gene expression between individuals with alternative QTL alleles, and identified an ADP-ribosylation factor (ARF) GTPase (*PtARF1*) as a candidate gene for regulating leaf morphology in this pedigree. ARF GTPases are critical elements in the vesicular trafficking machinery. Disruption of the vesicular trafficking function of ARF by the pharmacological agent Brefeldin A (BFA) altered leaf lateral growth in the narrow-leaf *P. trichocarpa* suggesting a molecular mechanism of leaf shape determination. Inhibition of the vesicular trafficking processes by BFA interferes with cycling of PIN proteins and causes their accumulation in intercellular compartments abolishing polar localization and disrupting normal auxin flux with potential effects on leaf expansion.

**Conclusions:**

In other model systems, ARF proteins have been shown to control the localization of auxin efflux carriers, which function to establish auxin gradients and apical-basal cell polarity in developing plant organs. Our results support a model where *PtARF1* transcript abundance changes the dynamics of endocytosis-mediated PIN localization in leaf cells, thus affecting lateral auxin flux and subsequently lamina leaf expansion. This suggests that evolution of differential cellular polarity plays a significant role in leaf morphological variation observed in subgenera of genus *Populus*.

**Electronic supplementary material:**

The online version of this article (doi:10.1186/s12870-015-0557-7) contains supplementary material, which is available to authorized users.

## Background

Leaf morphology has remarkable phenotypic diversity throughout the plant kingdom, making it a favorable system in which to study the evolution of variations in form. A number of genes and networks have been described that affect initial leaf development and pattern formation (reviewed thoroughly by [1] in both simple and complex leaves [2, 3]. Similarly, mutagenesis screens have identified genes that regulate leaf blade shape, primarily width [4–6], length [6–8] and their ratios [9]. An emerging paradigm from these discoveries is that two-dimensional leaf shape can be regulated both by differential cell elongation (polarity) or differential cell proliferation favoring one dimension versus the other [10]. This well-grounded knowledge of leaf initiation, development, and shape was established largely through mutagenesis of model systems. Comparably, little is known about whether these same molecular mechanisms underlie evolutionary differences in leaf morphological variation. Few studies have directly addressed whether alternative alleles at these genes underlie variation in leaf shape within or between different plant species [11]. In light of this shortcoming and the extensive diversity in leaf form, additional studies exploiting naturally occurring variation are needed to clarify the role of previously discovered genes in evolutionary variation for leaf traits and potentially discover new genetic regulators.

Quantitative trait locus-based approaches are frequently applied to uncover loci that regulate natural phenotypic diversity. Quantitative trait loci have been identified for leaf morphological traits in several species including tomato [12], poplar [13], oak [14], maize [15], and *Arabidopsis* [16]. These studies clearly show several aspects of leaf morphological variation to be subject to multigenic control. Thus, loci discovered on the basis of mutagenesis may only explain a portion of the naturally occurring variation in leaf shape. However, the molecular characterization of these regulatory loci discovered by QTL analysis has been generally prohibitive because of the challenges associated with moving from QTL to gene [17]. Novel experimental approaches developed from advances in genome sequencing and transcriptome analysis have eased QTL cloning [18–20], making QTL-based techniques powerful tools to elucidate molecular mechanisms underlying naturally occurring phenotypic variation [21, 22]. One approach to uncover genes underlying QTL is based on genetical genomics [18], which combines information from genome sequence with quantitative genetic analysis of gene expression and organismal traits of interest [23]. Transcriptome analysis extends the quantitative genetics (QTL) paradigm by providing information about an intermediate step between genotype and phenotype, potentially capturing not only genotypic but also developmental and environmental sources of variation. Genetical genomics was applied early on to model systems such as yeast [24] and *Arabidopsis* [25], and later to forestry crops to uncover genes involved in drough response [26] and lignin biosynthesis [27]. More recently the use of genetical genomics has expanded to several plant species, to understand the genetic regulation of developmental traits [28], as well as biotic [29] and abiotic [30] stress response.

The *Populus* genus is a particularly favorable system in which to apply a genetical genomics approach, given its extensive genetic and phenotypic variation, the availability of several well established interspecific pedigrees, and a rapidly growing genomic toolbox founded on the genome sequence of *P. trichocarpa* [31]. The genus is comprised of five evolutionary sections, and leaf morphological form is widely regarded as diagnostic of evolutionary relationships at the sectional level [32]. In addition, several studies have shown that leaf morphological characters are predictive of long-term clonal performance and growth [33–35]. Therefore, a detailed study of intersectional poplar hybrids may provide an approach to identify loci associated with this important phenotypic variation.

Here we analyze an intersectional pseudo-backcross pedigree of narrow-leaf *P. trichocarpa* Torr. & Gray (section *Tacamahaca*) and wide-leaved *Populus deltoides* Bartr. ex Marsh (section *Aigeiros*) for variation in leaf morphology using a genetical genomic approach to identify potential candidate genes as important regulators of leaf shape variation. Specially, we performed QTL analyses for leaf lamina width and lenth:width ratio in multiple experiments and a transcriptome analysis in expanding leaf tissue in this pedigree. An ADP-ribosylation factor (ARF) GTPase identified as a prime candidate gene for the regulation of leaf morphology in this pedigree was further fuctionally characterized using the pharmacological agent BFA, which interferes with the ARF GTP-GDP exchange [36, 37].

## Results

### Identification of a major QTL for leaf blade width

**Fig. 1:**
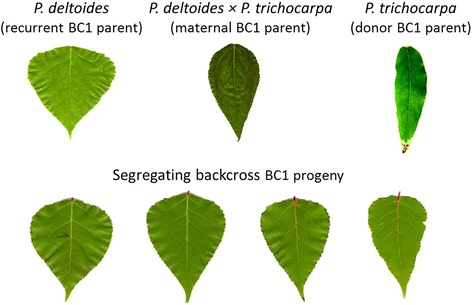
Leaf lamina shape variation among parents and progeny of the *P. trichocarpa* X *P. deltoides* pseudobackcross pedigree family 52–124. The donor parent, *P. trichocarpa*, has a lamina length/width ratio of ~3.0, while the recurrent parent, *P. deltoides*, has a lamina ratio of ~1.0. The trait exhibits additive variation, as the hybrid parent has a ratio of ~1.5. Segregating BC1 progeny span the spectrum of phenotypic varation from the hybrid to the recurrent parent with limited transgressive segregation observed

**Fig. 2:**
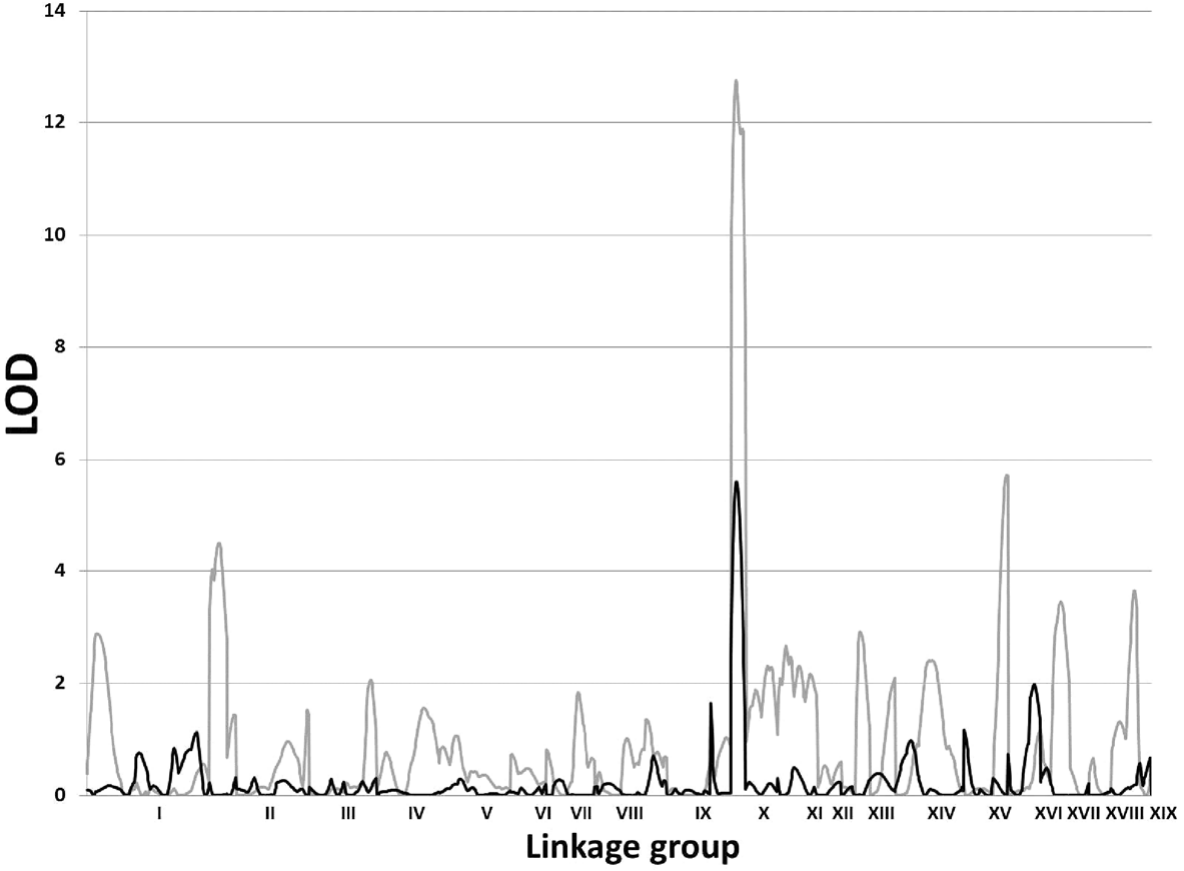
Genome-wide composite interval mapping scan for leaf lamina length/width ratio (grey line), and for the expression of *PtARF1* (black line) in family 52–124

We carried out a QTL analysis for variation in leaf lamina shape (measured by lamina length, width, and their ratio) in an interspecific pseudo-backcross pedigree (family 52–124) segregating between the narrow-leaf donor parent species *P. trichocarpa* and the broad-leaf recurrent parent *P. deltoides* (Fig. 1). One or more significant QTL were detected using composite interval mapping (CIM) [38, 39] using a standard threshold of the 95^th^ percentile of 1000 permutations, for all traits measured in the population of 396 individuals grown in a greenhouse at the University of Florida (Table 1). Additional analysis focused on a QTL on linkage group (LG) X that was most significantly associated with both lamina width and length:width ratio (Fig. 2). As the direction of the effects for these QTL were opposing – i.e., the same allele that increased lamina width also decreased lamina length:width ratio, we hypothesized that a common and pleiotropic regulator of leaf shape underlies the locus. Supporting this hypothesis, a negative genetic correlation was observed between these traits (*r*_G_ = −0.4628 ± 0.0653). The QTL interval, constrained by sequence-linked microsatellite markers PMGC_2855 and GCPM_2122, encompasses 625 genes and spans ~3.5 Mb of uninterrupted sequence in the genome sequence of *P. trichocarpa* [40]. To reduce the number of potential candidate genes in the leaf QTL interval, we increased mapping resolution by genotyping putative recombinant progeny (n = 96) for seven additional microsatellite markers identified from the genome sequence (Additional file 1). The additional microsatellites allowed us to decrease the sequence spanned by the QTL interval to ~3.0 Mb and 450 candidate genes (Additional file 2). Due to the high proportion of phenotypic variation explained and quality of the genome assembly in this QTL region, we elected to pursue the major locus on LG X for further characterization and QTL cloning.

**Table 1:** Summary of QTL detected for leaf lamina shape characters in *P. trichocarpa* X *P. deltoides* X *P. deltoides* family 52–124, in the population growth in greenhouse in Florida. Asterisks indicate QTL detected in a subset of the same population established in a greenhouse in Michigan

Trait	Clonal Repeatability (Std Error)	QTL #	Linkage group	Flanking Marker 1	Flanking Marker 2	Origin of positive allele	LOD peak	Phenotypic Variance Explained
Lamina Length	.2186 (.0244)	1	IV	G1809	G3847	*P. deltoides*	3.14	6.31 %
		2	XVI	rP2143	O533	*P. deltoides*	2.68^a^	8.1 %
Lamina Width	.2407 (.0249)	1	VI	P2221	W12	*P. trichocarpa*	3.69	5.12 %
		2	X	P2855	G2122	*P. deltoides*	4.59	5.99 %
Lamina Length:Width Ratio	.3490 (.0276)	1	I	G124	G2903	*P. deltoides*	4.51	6.16 %
		2	X	P2855	G2122	*P. trichocarpa*	12.77	14.20 %
		2	X	P2855	G2122	*P. trichocarpa*	4.49^b^	13.20 %
		3	XV	G1245	G1424	*P. deltoides*	5.73	5.12 %
		4	XVII	G3580	G641	*P. trichocarpa*	3.45	5.00 %
		5	XIX	O597	G2319	*P. deltoides*	3.69	4.00 %

^a^ Genome-wide significant LOD peak at Michigan Technological University using composite interval mapping with MAPQTL software

^b^ Chromosome-wide significant LOD peak at Michigan Technological University using composite interval mapping with MAPQTL software

### Trait and QTL stability in different experimental settings

QTL studies often present conflicting results when repeated across environments [41]. In order to assess the stability of the QTL detected initially, the study was repeated at Michigan Technological University by measuring variation in leaf lamina traits under controlled greenhouse conditions in 2010 and at a research field site in 2012. Composite interval mapping confirmed the QTL on linkage group X to be significantly associated with length:width ratio under greenhouse conditions (Table 1). However, the correlation between leaf traits measured under greenhouse and field conditions was low and not significant, suggesting highly plastic responses of the leaf traits to the field environment. Under field conditions no significant QTL was detected for any of the leaf traits.

### Genetical genomics identifies *PtARF1* as a candidate gene for lamina shape regulation

After decreasing the QTL interval we elected to move forward with a genetical genomics approach that integrates quantitative genetic analysis of phenotypes and gene expression, to further reduce the pool of candidate genes. Because of the significant role of gene expression in evolutionary diversification of species [42], we hypothesized that interspecific differences in transcription of a key regulator of cell division or expansion may explain the leaf lamina variation observed in the progeny of family 52–124. Thus, we identified genes that are differentially regulated in developing leaves of family 52–124, located within the major QTL interval in LG X, and *cis*-regulated by the same locus. We measured genome-wide gene expression in expanding leaf tissue from one biological replicate of each of 183 segregating progeny using a microarray that included probes for all gene models annotated from the *P. trichocarpa* genome. The normalized transcript data was mapped as expression quantitative trait loci (eQTL). This genome-wide analysis identified 13,403 statistically significant eQTL representing 12,392 unique gene models, as described previously [43].

Since our hypothesis was that lamina shape variation was a product of differential gene regulation arising from interspecific polymorphism(s) within the leaf QTL interval, we narrowed our focus to the genes with eQTL surpassing the significance threshold in this region. Our analysis identified 161 eQTL, which we classified as *cis*- (n = 116) or *trans*- (n = 45) acting, contingent on the physical position of the gene model in the genome assembly. We classified all eQTL arising from gene models genomic scaffolds (n = 19) that were not anchored to the genome sequence as *trans*-acting, since the *Populus* genome sequence assembly in the leaf trait QTL region was predicted to be contiguous [31].

We expected that if differential transcript accumulation of a key regulatory factor(s) accounts for phenotypic variation in leaf shape, abundance of such transcripts should exhibit a significant statistical correlation with leaf lamina shape. Furthermore, as differential regulation of these transcripts influences variation in the phenotypes, they should have eQTL co-localized in the leaf QTL interval. Thus, we utilized a standard multivariate correlation to determine the relationship between the leaf lamina phenotypes and each transcript with an eQTL in the leaf trait QTL interval. Among the 161 transcripts with eQTL co-localized to the trait interval, we identified 25 correlated (|*r*| > 0.20) to either lamina width, length:width ratio, or both (Table 2). Among these 25 genes, only two were correlated to both measures of lamina shape at |*r*| > .20. Furthermore, only Potri.010G152600 was regulated in *cis-* by the trait QTL region (Fig. 2), indicating that polymorphisms within its regulatory region affects its transcript level (eQTL) and might be the cause for the phenotypic variation controlled by the QTL on linkage group X.

**Table 2:** Phenotypic-expression correlation for 25 genes with significant cis- and trans-eQTL regulated by the major lamina shape QTL locus on linkage group X

*P. trichocarpa* Gene Model	*cis/trans* eQTL	Correlation Lamina Length	Correlation Lamina Width	Correlation Lamina Length/Width Ratio	Ath Ortholog	Ath Ortholog Annotation
Potri.010G152600	*cis*	−0.2354195	−0.3668554	0.25168267	AT1G10630	ATARFA1F; GTP binding/phospholipase activator/protein binding
estExt_Genewise1_v1.C_LG_X0900	*cis*	0.28153168	0.22276079	0.12428305	AT2G03420	similar to expressed protein
estExt_Genewise1_v1.C_LG_X1905	*cis*	−0.1180535	−0.2092655	0.16315263	AT5G05840	similar to unknown protein
Potri.010G146900	*cis*	−0.2057423	−0.2864154	0.15937637	AT5G49480	ATCP1 (CA2 + −BINDING PROTEIN 1); calcium ion binding
Potri.010G144500	*cis*	−0.1709655	−0.2330248	0.09520016	AT3G66654	peptidyl-prolyl cis-trans isomerase cyclophilin-type family protein
Potri.010G115500	*cis*	−0.1655205	−0.239631	0.13854495	AT1G71110	similar to unknown protein
eugene3.00101684	*cis*	−0.0327462	−0.1340572	0.22250948	AT1G79270	ECT8 (evolutionarily conserved C-terminal region 8)
eugene3.00101873	*cis*	0.13606338	0.00904373	0.27538385	AT2G25770	similar to unknown protein
Potri.010G085300	*trans*	−0.1625601	−0.2031862	0.095351	AT3G22600	protease inhibitor/seed storage/lipid transfer protein (LTP) family protein
fgenesh4_pg.C_LG_X001101	*cis*	−0.1932028	−0.2426184	0.10447822	AT1G18390	protein kinase family protein
fgenesh4_pg.C_LG_X001267	*cis*	−0.2074094	−0.2653902	0.10578893	AT3G14630	CYP72A9 (cytochrome P450)
Potri.010G120500	*cis*	0.27906541	0.1749712	0.21085259	AT1G68220	similar to unknown protein
grail3.0022009901	*cis*	0.27753008	0.20094372	0.15049532	AT4G00170	vesicle-associated membrane family protein/VAMP family protein
Potri.010G189300	*cis*	−0.0907817	−0.1946685	0.22753633	AT3G11210	GDSL-motif lipase/hydrolase family protein
gw1.X.1611.1	*cis*	−0.0336113	−0.1425979	0.24313545	AT5G05460	hydrolase
Potri.010G184700	*cis*	0.03487584	−0.0635483	0.25117987	AT4G35920	MCA1 (MID1-COMPLEMENTING ACTIVITY 1)
gw1.X.2067.1	*cis*	0.20184048	0.06762258	0.30451177	AT3G11420	fringe-related protein
Potri.010G119300	*cis*	0.18423729	0.21675814	−0.0307313	AT1G15080	LPP2
gw1.X.558.1	*cis*	−0.2016639	−0.2470379	0.10097016	AT4G02080	ATSAR2
gw1.X.613.1	*cis*	−0.152675	−0.2248164	0.13504918	AT1G56430	nicotianamine synthase
Potri.010G149500	*cis*	0.17168568	0.0563584	0.20768151	AT3G06510	SFR2 (SENSITIVE TO FREEZING 2); hydrolase
Potri.010G179600	*trans*	0.01835356	0.14446327	−0.246553	AT1G65470	FAS1
eugene3.212260001	*trans*	−0.2032587	−0.2235336	−0.0071332	AT4G27570	glycosyltransferase family protein
fgenesh4_pg.C_scaffold_44000104	*trans*	−0.0999934	−0.205086	0.29000845		No Hits
fgenesh1_pg.C_LG_IX000767	*trans*	−0.183402	−0.2330726	0.09782444		No Hits

The closest homolog of Potri.010G152600 in *Arabidopsis thaliana* is At2g47170, (e-value = 2e-99, 96 % amino acid sequence identity), an ADP-ribosylation factor (ARF) -type GTPase (ARF1). Thus, the predicted gene Potri.010G152600 was renamed *PtARF1. PtARF1* is located in close proximity to the leaf QTL logarithm of the odds (LOD) peak positioned at 98.11 cM of linkage group X. The 2 centimorgan (cM) region (97.11 cM–99.11 cM) with the highest LOD score spans 285.1 Kbp (from 15819851–16104972 on scaffold 10) and contains 24 candiate genes with putative function or known amino acid domains, of which *PtARF1* is located at position 16037970.

### Disruption of ARF function leads to alteration in leaf width growth

Plant ARFs and ARF GEFs (guanine-nucleotide exchange factors for ADP-ribosylation factor GTPases) are essential for vesicular trafficking in all eukaryotic kingdoms. ARF1-GTP has higher affinity for membranes and is essential for the formation of the budding vesicle. This mechanism has been dissected extensivelly using the pharmacological agent BFA, which interferes with the ARF GTP-GDP exchange [36, 37]. In plants treated with BFA, PIN accumulates in intracellular compartments, abolishing polar localization and disrupting normal auxin flux [44, 45].

To begin addressing the role of poplar ARFs in vesicle trafficking and PIN distribution in leaf development we established cuttings of the narrow-leaf *P. trichocarpa* in a hydroponic solution and treated them with BFA. After 48 h in ½ Murashige and Skoog solution, plants were transferred to the same nutrient solution supplemented with 0, 5 and 10 μM of BFA. The length and width of the first fully unfolded leaf (leaf plastochron index [LPI] 2) was recorded at the time of transfer and again 24 h after cuttings were transferred to the BFA-containing hydroponic solution. Within this interval, we observed an increase in the ratio of length to width of +0.08 (standard deviation, s.d = 0.06) in the control conditions, which is commonly observed in *P. trichocarpa* leaves undergoing expansion. Contrastingly, there was a significant decrease in the ratio of length to width of −0.13 (s.d. = 0.11, *p*-value = 0.01) and −0.18 (s.d. = 0.21, *p*-value < 0.05) in plants treated with 5 and 10 μM of BFA, respectively. Measurements were not made for a longer period of time because development was significantly impaired in treated plants compared to controls. These results suggest that ARF-mediated vesicle trafficking and PIN localization are critical determinants of leaf morphology in *Populus*.

## Discussion

A key goal of quantitative genetics is to move from a phenotypic QTL to the polymorphisms that regulate it. Evidence in model systems has shown that genetical genomics, which allies the quantitative analysis of phenotypes and gene expression data in segregating populations, can effectively identify regulatory genes [18–20]. Here, we considered leaf lamina shape among an interspecific hybrid progeny of *Populus* in a genetical genomics context. From a series of QTL for leaf lamina shape characters, we identified a major QTL implicated in lamina morphology – pleiotropically regulating both lamina width and lamina length:width ratio.

The major QTL for leaf length:width ratio of linkage group X was consistently identified in two greenhouse experiments, confirming it as a major effector for leaf lamina shape variation in a controlled physical environment and an attractive candidate for downstream characterization. However, the QTL was not identified under field conditions suggesting that expression of the trait is plastic and potentially affected by microenvironmental variations including effects of soil, water availability and temperature. Similar to our observation, in an interspecific willow progeny, QTL positions varied for growth phenology traits between controlled and field conditions reflecting the effects of different environments [46]. In a previous QTL study, we have demonstrated that a simple change in nitrogen availability completely changes the genetic control of biomass growth and allocation, as well as carbon partitioning within wood components [62]. Phenotypic plasticity, mainly due to variation in temperature, has also been reported to affect bud set traits in black poplar planted at two sites with contrasting environmental conditions [47].

Utilizing whole-genome microarrays for expression analysis of leaf tissue, we identified a small group of genes whose expression was statistically correlated with the phenotypes of interest and whose transcript abundances were *cis*-regulated by the phenotypic QTL interval. Considering the statistical relationships with leaf morphology traits, genetic regulation of expression, and functional annotations, an ADP-ribosylation factor GTPase – *PtARF1* – was identified as a prime candidate gene governing lamina shape characters in the interspecific hybrid pedigree. Few of the other flanking genes close to the QTL maximum have been previously reported to be involved in leaf development and other processes. For example, the Myb-like DNA-binding domain controls leaf cell differentiation in *Arabidopsis* [48]. This gene is also responsible for longer and narrower leaves in maize [49]. However, regulation of expression (eQTL) in none of these flanking genes co-located with the major QTL.

ARF genes are implicated in vesicle trafficking – a critical process for cell polarity during development of yeast, animals and plants. The hormone auxin regulates many aspects of plant development [50, 51]. Auxin distribution in cells is mediated by PIN, which determines the direction of auxin intercellular flow and, therefore, influences plant growth and development [52]. It has been previously shown that vesicle trafficking is essential for the localization of PIN. For proper localization, PIN must cycle between the plasma membrane and the endosomal compartments [53]. Therefore, because the polar transport of auxin mediates various essential processes in plant growth, vesicle trafficking is critical for development. In *Arabidopsis* mutants for a known component of the vesicle trafficking machinery, the ADP-ribosylation factor GTP exchange factor (GNOM ARF-GEF), PIN localization was disrupted resulting in loss of cell alignment in the embryonic axis [54, 55]. PIN proteins are post-translationally localized to apical and basal membranes of *Arabidopsis* epidermal cells in an endocytic process mediated by two Rab5 GTPase homologs [53]. Similarly, the action of *AtARF1* (the ortholog of *PtARF1*) has also been shown to modulate the kinetics of endocytosis and PIN2 localization, as well as cell polarity, in *Arabidopsis* roots [44]. The connection between cell polarity and auxin has been intricately studied in several model plant systems [56, 57]. Auxin has already been implicated in initial leaf formation [58, 59], lamina margin elaboration [2, 3] and leaf vasculature patterning [60]. Thus, it is likely that leaf expansion is also directly affected by auxin flux. In *Populus*, our evidence supports a model whereby differential expression of *PtARF1* in *P. trichocarpa* and *P. deltoides* changes the dynamics of endocytosis-mediated PIN localization in leaf cells. Decreased abundance of *ARF1* transcript, and hence, ARF1 protein in *P. deltoides* slows the process of PIN polarization through the endocytic pathway during development and expansion. Decreased PIN polarization results in increased lateral auxin flux and, subsequently, increased lamina expansion in the leaf width direction. Conversely, in *P. trichocarpa*, higher abundance of *ARF1* transcript leads to a more abundant supply of ARF1 protein, which increases the relative rate of endocytosis and PIN polarization. More rapid PIN polarization increases auxin flux and expansion of the lamina in the leaf length direction, decreasing lateral leaf expansion.

## Conclusion

Collectively, our results provide compelling evidence for the role of *PtARF1* in shaping variations in leaf morphology. Similarly, we provide another possible way in which the key plant hormone auxin could shape diversity in plant morphology. We have demonstrated that natural variation for auxin response might have a clear role in plant diversity we observe in nature, and our analysis suggests that natural variation in hormone response pathways clearly warrants additional investigation as we learn more about the evolution of morphological differences among plant taxa.

## Methods

### Plant material and phenotyping

This study was carried out in a pseudo-backcross pedigree of *P. trichocarpa* × *P. deltoides* (clone 52–225) and *P. deltoides* (clone D-124), referred hereafter as family 52–124. This pedigree was previously genotyped, phenotyped and quantitatively analyzed for several growth and developmental traits measured in plants grown in a greenhouse at the University of Florida (Gainesville, Florida, USA) in the spring of 2006 [61, 62]. In the present study, leaf morphology was measured digitally using Image Pro Plus software (Media Cybernetics, Inc., Bethesda, MD) from image scans of the leaf closest to one-half the live crown height, in three biological replicates of 396 individuals. Traits measured included leaf lamina length, width, and their ratio. Leaf length was measured along the midvein from the junction of the lamina and petiole, to the distal tip of the leaf. Blade width was measured at the widest point of the lamina. An analysis of variance [62] was applied to the phenotypic measurements to generate a least-square mean genotypic value for each individual that was used for subsequent QTL analysis. Clonal repeatability and genetic correlations were calculated as described previously [62], except excluding the effects of row and column position in the experimental design.

To evaluate the stability of QTL expression in another experimental setting, a subset of 153 individuals from family 52–124 were grown in a greenhouse at Michigan Technological University (Houghton, Michigan, USA) in the spring of 2010. Cuttings were rooted with rootone (Garden Tech) and grown in one-gallon pots in sunshine mix 1 potting soil containing 70-80 % Canadian Sphagnum peat moss, perlite, dolomite limestone, Gypsum and wetting agent (Sun Grow Horticulture, Canada). Leaf morphological traits were assessed on the three largest fully expanded leaves of the leading shoot for each individual as described above. Mean values were calculated for each genotype for subsequent QTL analysis. Plants were transferred to a field site (2 m × 2 m spacing) in June 2011 at the Ford Center Research Forest, south of L’Anse, Michigan (46°38′37″N 88°28′46″W), and leaf morphological traits were scored again in summer of 2012. Correlation among leaf traits and years was calculated using Pearson’s correlation coefficients.

### Genotyping and genetic mapping of progeny

A previously developed microsatellite and microarrray-based genetic map [62] that covers at least 85 % of the reference *P. trichocarpa* genome was used for QTL mapping of the leaf traits. Additional microsatellite markers within the primary QTL interval were identified from the 'Nisqually-1' genome sequence (Additional file 1, [40]) using MsatFinder v.2.0 software [63]. Primers were designed for these loci within the MsatFinder interface, and loci were amplified and genotyped in 96 recombinant progeny (as judged by flanking framework markers), using 1 % agarose gels (w/v) as described in a previous study [61].

### Leaf trait QTL analysis

QTL for leaf traits measured at the University of Florida were initially identified in QTL Cartographer v.4.0 [65], using CIM [38, 39] with a standard threshold of the 95^th^ percentile of 1000 permutations. QTL for leaf traits assessed at Michigan Technological University were identified using MAPQTL v.5.0 [66, 67] using the same method (CIM) and statistical threshold applied in the University of Florida experiment. The maximum LOD and genome-wide/chromosome-wide significance intervals were determined for each QTL. Putative candidate genes underlying these QTL intervals were identified using the genome sequence of *P. trichocarpa* available at Phytozome v. 9 [68].

### Transcriptome analysis

RNA was isolated immediately after collection [64] from one leaf apical and basal to the phenotyped leaf, in one biological replicate of 183 individuals grown in a greenhouse at the University of Florida. RNA was converted to double-stranded complementary DNA, labeled, and hybridized to a customized four-plex NimbleGen microarray platform (Gene Expression Omnibus Accession# GPL7234) using probes that minimize the contribution of sequence polymorphism to estimates of gene expression. The microarray included 55,793 60-mer probes, including one for each of the previously described gene models derived from the annotation of the genome sequence of *P. trichocarpa* clone 'Nisqually-1', and a set of non-annotated ESTs. The RNA manipulation methods and microarray design have been previously described in detail [61]. The raw data from hybridizations were background subtracted, log_2_ transformed, and quantile normalized. The data is publically available (Gene Expression Omnibus Accession# GSE12623, GSE20117 and GSE20118) and was used as input for gene expression QTL analysis.

### Gene expression QTL analysis

Gene expression QTLs were identified, measured for significance, and classified as *cis*- or *trans*-acting as described previously [43]. Briefly, the quantile-normalized gene expression was analyzed with CIM implemented in QTL Cartographer [65], using the genetic map of family 52–124 [61]. Expression QTLs were declared to be significant based on a global permutation threshold of 95 % and classified as *cis*- or *trans*-regulated based on them being co-localized within the same marker interval where the respective gene model was located in the 'Nisqually-1' genome sequence.

To determine the relationship between gene expression and phenotype, phenotypic values from the same biological replicate measured for gene expression were correlated with the normalized gene expression values using the multivariate Pearson correlation function of JMP 7.0 (SAS Institute, Cary, NC). Finally, trait QTL position was confirmed for the single biological replicate on which gene expression was measured, using the same approach described above.

### *In vivo* characterization of ARF function for leaf lamina shape determination

A set of 36 cuttings (6–7 cm) were collected from the *P. trichocarpa* Nisqually-1 reference genotype (narrow leaf) and grown in hydroponic chambers containing ½ concentration MS solution for 48 h. Next, cuttings were transferred to the same nutrient solution supplemented with 0, 5 and 10 μM of BFA, which inhibits vesiclular trafficking. A total of 12 cuttings were used in each treatment. Length and width of the first fully unfolded leaf (leaf plastochron index [LPI]) were recorded at time 0 (when cuttings were transferred to the BFA-containing hydroponic solution) and 24 h later. Measurements were not made for a longer period of time because development was significantly impaired in treated plants compared to controls. The distribution of length to width ratio values measured in the 12 biological replicates, in plants treated with BFA (5 and 10 μM) was contrasted with values observed in the non-treated plants using a two-sample *t*-test, and *p*-values were estimated.

### Availability of supporting data

The microarray data is publically available in the National Center for Biotechnology Information Gene Expression Omnibus under the accession numbers GSE12623, GSE20117 and GSE20118.

## Additional files

Additional file 1:
**Title of data: Primer list.** Description of data: Primers utilized to amplify microsatellite loci across the major linkage group X QTL for leaf lamina characters in family 52–124. Additional file 2:
**Title of data: Fine-scale lead and expression QTL mapping.** Description of data: Fine-scale mapping of the major lamina shape QTL on LG X in the segregating pedigree. Framework SSR loci were genotyped in 396 progeny (“G_” and “P_” loci) while additional SSR underlying the QTL were genotyped in 96 recombinant progeny. Recombinants were identified by maternally inherited marker genotypes at locus P_2855 and G_2122.

## Abbreviations

ARF: ADP-ribosylation factor
BFA: Brefeldin A
CIM: Composite interval mapping
cM: Centimorgan
eQTL: Expression quantitative trait locus
GEF: Guanine-nucleotide exchange factors
LF: Linkage group
LOD: Logarithm of the odds
QTL: Quantitative trait locus
