# Supplementary material for: Genetical genomics of Populus leaf shape variation
Source: BMC Plant Biol. 2015 Jun 30;15:166. doi: 10.1186/s12870-015-0557-7 (PMC4486686; doi:10.1186/s12870-015-0557-7)
Supplement: Additional file 1: — Title of data: Primer list. Description of data: Primers utilized to amplify microsatellite loci across the major linkage group X QTL for leaf lamina characters in family 52–124. [file 12870_2015_557_MOESM1_ESM.docx]

Additional file 1. Primers utilized to amplify microsatellite loci across the major linkage group X QTL for leaf lamina characters in family 52-124.

| Locus | Primer Forward Sequence | Primer Reverse Sequence | Motif | Repeats | Linkage Group X Physical Location (bp) |
| --- | --- | --- | --- | --- | --- |
| PMGC_2855 | GGTATCTTGTTATCCACTGCC | TTTTCCTCGTTAATTAGAGTCG | GA | n/a | 11122293 |
| LG_X01 | GCCACCAATCCAGCAAGTAA | AATGGAGGTGTGGCAGTAGC | TA | 9 | 11872486 |
| LG_X06 | GAGAAGCAGCAATGCAGGAT | ATGCAAACTGGTCCGGATAC | CTT | 6 | 12645373 |
| LG_X03 | ACCTGGTCCATTTGTTGAGC | TGCAGGCAATCTCAAACTCA | GA | 5 | 13378326 |
| LG_X04 | GGGATGGCAAAATACGTTCA | CATCATCGTACAACCTCACCTT | TA | 5 | 14126639 |
| ARF1US3 | GGCAAGGACAGCGCATGATG | TTTCACCGAACTTCCACACTTT | n/a | n/a | 14365406 |
| ARF1DS2 | GTGATAGCAGAGAGCCGAAA | GTAAGTATGAGAGGAATGAGGGG | n/a | n/a | 14369387 |
| LG_X05 | TGGTAGATTGCGAGCTGAGA | AGGGCTTTCCGGCTATTAAA | CT | 5 | 15159499 |
| GCPM_2122 | TCAGCAACTATCACCATGAA | GGAATGTGCAGCATATACAA | GT | 12 | 15661771 |
