# Supplementary material for: Genetical genomics of Populus leaf shape variation
Source: BMC Plant Biol. 2015 Jun 30;15:166. doi: 10.1186/s12870-015-0557-7 (PMC4486686; doi:10.1186/s12870-015-0557-7)
Supplement: Additional file 2: — Title of data: Fine-scale lead and expression QTL mapping. Description of data: Fine-scale mapping of the major lamina shape QTL on LG X in the segregating pedigree. Framework SSR loci were genotyped in 396 progeny (“G_” and “P_” loci) while additional SSR underlying the QTL were genotyped in 96 recombinant progeny. Recombinants were identified by maternally inherited marker genotypes at locus P_2855 and G_2122. [file 12870_2015_557_MOESM2_ESM.docx]

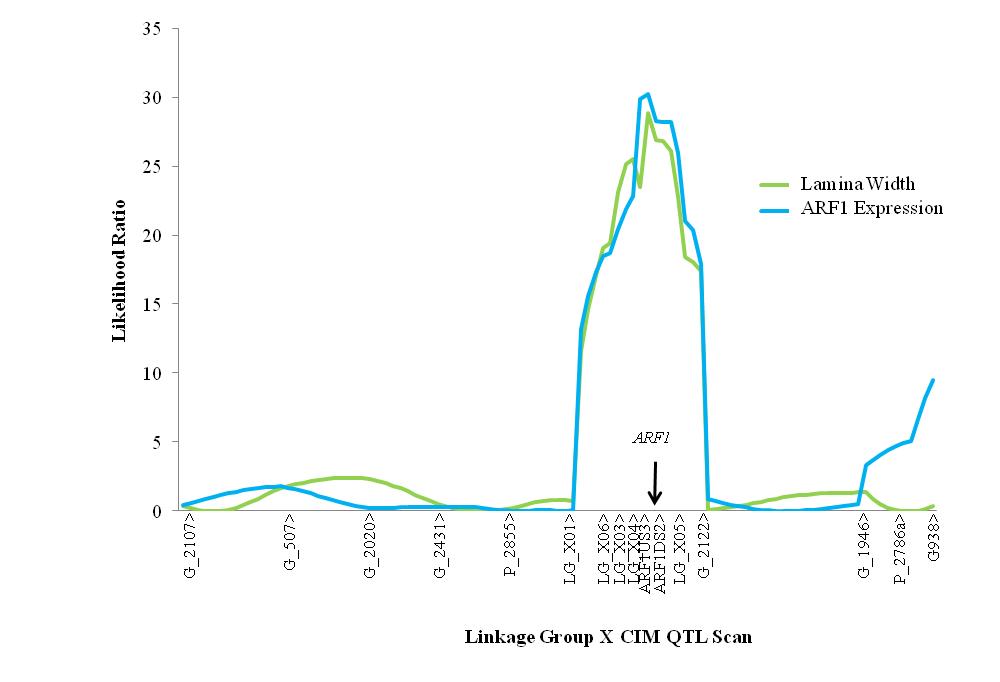


Additional file 2**.** Fine-scale mapping of the major lamina shape QTL on LG X in the segregating pedigree. Framework SSR loci were genotyped in 396 progeny (“G_” and “P_” loci) while additional SSR underlying the QTL were genotyped in 96 recombinant progeny. Recombinants were identified by maternally inherited marker genotypes at locus P_2855 and G_2122.
